# Cross-species alcohol dependence-associated gene networks: Co-analysis of mouse brain gene expression and human genome-wide association data

**DOI:** 10.1371/journal.pone.0202063

**Published:** 2019-04-24

**Authors:** Kristin M. Mignogna, Silviu A. Bacanu, Brien P. Riley, Aaron R. Wolen, Michael F. Miles

**Affiliations:** 1 Virginia Institute for Psychiatric and Behavioral Genetics, Virginia Commonwealth University, Richmond, Virginia, United States of America; 2 VCU Alcohol Research Center, Virginia Commonwealth University, Richmond, Virginia, United States of America; 3 VCU Center for Clinical & Translational Research, Virginia Commonwealth University, Richmond, Virginia, United States of America; 4 Department of Human and Molecular Genetics, Virginia Commonwealth University, Richmond, Virginia, United States of America; 5 Department of Pharmacology and Toxicology, Virginia Commonwealth University, Richmond, Virginia, United States of America; Oregon Health and Science University, UNITED STATES

## Abstract

Genome-wide association studies on alcohol dependence, by themselves, have yet to account for the estimated heritability of the disorder and provide incomplete mechanistic understanding of this complex trait. Integrating brain ethanol-responsive gene expression networks from model organisms with human genetic data on alcohol dependence could aid in identifying dependence-associated genes and functional networks in which they are involved. This study used a modification of the Edge-Weighted Dense Module Searching for genome-wide association studies (EW-dmGWAS) approach to co-analyze whole-genome gene expression data from ethanol-exposed mouse brain tissue, human protein-protein interaction databases and alcohol dependence-related genome-wide association studies. Results revealed novel ethanol-responsive and alcohol dependence-associated gene networks in prefrontal cortex, nucleus accumbens, and ventral tegmental area. Three of these networks were overrepresented with genome-wide association signals from an independent dataset. These networks were significantly overrepresented for gene ontology categories involving several mechanisms, including actin filament-based activity, transcript regulation, Wnt and Syndecan-mediated signaling, and ubiquitination. Together, these studies provide novel insight for brain mechanisms contributing to alcohol dependence.

## Introduction

Alcohol Use Disorder [1], which spans the spectrum from abusive drinking to full alcohol dependence (AD), has a lifetime prevalence of 29.1% among adults in the United States [2]. Alcohol misuse ranks third in preventable causes of death in the U.S. [3] and fifth in risk factors for premature death and disability, globally [4]. Although pharmacological therapy for AUD exists [5], the effectiveness is limited and the relapse rate is high. Improvement in AUD treatment requires research on the underlying genetic and biological mechanisms of the progression from initial exposure to misuse, and finally to dependence.

Twin studies estimate that AUD is roughly 50% heritable [6, 7]. Multiple rodent model studies have used selective breeding to enrich for ethanol behavioral phenotypes or have identified ethanol-related behavioral quantitative trait loci [8–10], further confirming the large genetic contribution to alcohol behaviors. Recent studies have also documented genetic factors influencing the effectiveness of existing pharmacological treatments for AD, further substantiating genetic contributions to the mechanisms and treatment of AUD [11]. Genome-wide association studies (GWAS) in humans have identified several genetic variants associated with alcohol use and dependence [12–15]. However, they have yet to account for a large portion of the heritability estimated by twin studies. Lack of power, due to a large number of variants with small effects, is believed to the source of this “missing heritability”^”^ [16]. Although recent large-scale studies have shown promise in identifying novel genetic contributions to alcohol consumption, these studies do not contain the deep phenotypic information necessary for identifying variants associated with dependence [14, 15]. Further, such GWAS results still generally lack information about how detected single gene variants are mechanistically related to the disease phenotype.

Genome-wide gene expression studies are capable of improving the power of GWAS by providing information about the gene networks and biological mechanisms in which GWAS variants function [17–20]. Although gene expression in brain tissue has been studied in AD humans [17, 18], these studies are often difficult to conduct and interpret, due to lack of control over experimental variables and small sample sizes. However, studies have found high conservation in gene expression correlation patterns between mice and humans, particularly in brain tissue [21]. Furthermore, extensive studies in rodent models have successfully identified ethanol-associated gene expression differences and gene networks in brain tissue [22–26]. Multiple ethanol-behavioral rodent models exist to measure different aspects of the developmental trajectory from initial exposure to compulsive consumption [27]. Acute administration to naïve mice models the response of initial alcohol exposure in humans, which is an important predictor of risk for AD [28, 29]. Wolen et al. used microarray analysis across a mouse genetic panel to identify expression correlation-based networks of acute ethanol-responsive genes (genes whose expression levels change after ethanol consumption or treatment), along with significantly associated expression quantitative trait loci in brain regions subserving the mesolimbocortical dopamine reward pathway—the prefrontal cortex (PFC), nucleus accumbens (NAc), and ventral tegmental area (VTA) [25]. Furthermore, specific networks also correlated with ethanol behavioral data derived from the same mouse genetic panel (BXD recombinant inbred lines) [10]. Importantly, these gene expression responses to acute ethanol in BXD mice were later shown by our laboratory to have highly significant overlap with expression responses in a chronic ethanol exposure model known to mimic aspects of alcohol dependence in humans [30], and also contained a gene expression network associated with alcohol dependence thatwe recently identified *Gsk3b* as a potential candidate gene for treatment of alcoholism [31]. Together, these results support our premise that acute ethanol-exposed rodent brain gene expression could provide insight into relevant mechanistic frameworks and pathways underlying ethanol behaviors and risk for dependence in humans.

Several studies have integrated GWAS and gene expression or gene network data to cross-validate behavioral genetic findings [17]. For instance, the Psychiatric Genomics Consortium [32] tested for enrichment of nominally significant genes from human GWAS in previously identified functional pathways, and found shared functional enrichment of signals for schizophrenia, major depression disorder, and bipolar disorder in several categories. These pathways included histone methylation, neural signaling, and immune pathways [32]. Mamdani et al. reversed this type of analysis by testing for significant enrichment of previously identified GWAS signals in gene networks from their study. They found that expression quantitative trait loci for AD-associated gene expression networks in human nucleus accumbens tissue had significant enrichment with AD diagnosis and symptom count GWAS signals from the Collaborative Study on the Genetics of Alcoholism dataset [17]. Additional approaches have taken human GWAS significant (or suggestive) results for AD and provided additional confirmation by showing that expression levels for such genes showed correlations with ethanol behaviors in rodent models [33]. Such methods are informative with respect to analyzing the function of genes that have already reached some association significance threshold. However, they do not provide information about genes not reaching such statistical thresholds, but possibly still having important contributions to the genetic risk and mechanisms of AUD.

Dense module searching for GWAS (dmGWAS) is an algorithm for directly integrating GWAS data and other biological network information so as to identify gene networks contributing to a genetic disorder, even if few of the individual network genes exceed genome-wide statistical association thresholds [34]. The initial description of this approach utilized Protein-Protein Interaction (PPI) network data to identify networks associated with a GWAS phenotype. Modules derived from protein-protein interactions were scored from node-weights based on gene-level GWAS *p*-values. This approach was used to identify AD-associated PPI networks that replicated across ethnicities and showed significant aggregate AD-association in independent GWAS datasets [35], thus demonstrating the potential utility of the method. A more recent iteration of the dmGWAS algorithm, termed Edge-Weighted dense module searching for GWAS (EW-dmGWAS), allows integration of gene expression data to provide a direct co-analysis of gene expression, PPI, and GWAS data [36].

Utilization of the EW-dmGWAS algorithm would allow for identification of gene networks coordinately weighted for GWAS significance for AD in humans and ethanol-responsiveness in model organism brain gene expression data. We hypothesized that such an approach could provide novel information about candidate gene networks likely contributing to the genetic risk for AUD, while also adding mechanistic information about the role of such networks in ethanol behaviors. We show here the first use of such an approach for the integration of human PPI connectivity with mouse brain expression responses to acute ethanol and human GWAS results on AD. Our design incorporated the genome-wide microarray expression dataset derived from the acute ethanol-exposed mouse brain tissue used in Wolen et al. [10, 25], human protein-protein interaction data from the Protein Interaction Network database, and AD GWAS summary statistics from the Irish Affected Sib-Pair Study of Alcohol Dependence [33]. Importantly, we validated the identified ethanol-responsive and AD-associated networks by co-analysis with an additional, independent AD GWAS study on the Avon Longitudinal Study of Parents and Children dataset. Our results, although requiring further detailed investigation, could provide important methodological and biological function insight for future studies on the mechanisms and treatment of AUD.

## Materials and methods

### Samples

#### Mouse gene expression data

In order to maximize the utility of the mouse model by minimizing effects of behavioral and environmental variation, and to afford the identification of dependence-contributing genes involved in initial ethanol response, this study utilized gene expression data from ethanol-naïve mice treated with a single dose of acute ethanol. All mouse brain microarray data (Affymetrix GeneChip Mouse Genome 430 2.0) are from Wolen et al., 2012 [25] and can be downloaded from the GeneNetwork resource (www.genenetwork.org), via accession numbers GN135-137, GN154-156 and GN228-230, respectively for PFC, NAc and VTA data. Treatment and control groups each contained one sample (pooled RNA from 3 mice) from each strain and were given IP injections of saline or 1.8 g/kg of ethanol, respectively. Euthanasia and brain tissue collection took place 4 hours later. Data used for edge weighting in EW-dmGWAS analysis included Robust Multi-array Average (RMA) values, background-corrected and normalized measures of probe-wise expression, from the PFC, VTA, and NAc of male mice in 27–35 BXD recombinant inbred strains and two progenitor strains (DBA/2J and C57BL/6J).

**Fig 1:**
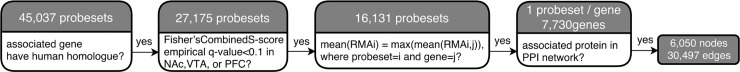
Data pipeline for determining ethanol-responsiveness and merging datasets. Pipeline used to prepare the data for the present analysis. The first cell contains the starting number of genes in the BXD mouse PFC, NAc, and VTA gene expression dataset.

Ethanol-responsive genes are predicted to be involved in pathways of neural adaptations that lead to dependence [25]. We predicted they would also be involved in mechanistic pathways from which GWAS signals are being detected. We therefore filtered for ethanol-responsive gene expression as done in Wolen et al.[25] prior to EW-dmGWAS so as to ensure edge weighting focused on ethanol responsiveness. Probe-level expression differences between control and ethanol-treated groups using the S-score algorithm which performs a probe-level analysis of expression between two groups [23, 37, 38] were obtained from the Wolen study [25] (S1 Table). Fisher’s Combined Test determined S-score significance values for ethanol responsiveness of each probeset across the entire BXD panel, and empirical p-values were calculated by 1,000 random permutations. Finally, q-values were calculated from empirical p-values to correct for multiple testing[25]. We defined an ethanol responsive gene set using a S-score probeset-level threshold of *q*_*FDR*_<0.1 for differential expression, in any one of the three brain regions. Genes associated with these probesets were carried forward in our analysis (Fig 1). Multiple probesets from single genes were reduced to single gene-wise expression levels within a particular brain region by selecting the maximum brain region-specific RMA value for each gene. After removing genes that were absent from the human datasets, 6,050 genes remained with expression values across all three brain regions (Fig 1).

#### Human GWAS data

Although many GWAS datasets now exist for AD, alcohol consumption and other ethanol responses, we chose two AD-related datasets for our analysis because of the phenotypic and methodological similarity between the studies and their availability at the time this work was initiated. The Irish Affected Sib-Pair Study of Alcohol Dependence (IASPSAD) AD GWAS dataset was used for the EW-dmGWAS analysis. It contains information from 1,748 unscreened controls (43.2% male) and 706 probands and affected siblings (65.7% male) from a native Irish population, after quality control [33]. Samples were genotyped on Affymetrix v6.0 SNP arrays. Diagnostic criteria for AD were based on the DSM-IV, and probands were ascertained from in- and out-patient alcoholism treatment facilities. Association of each Single Nucleotide Polymorphisms (SNP) with AD diagnosis status was tested by the Modified Quasi-Likelihood Score method [39], which accounts for participant relatedness. SNPs were imputed using IMPUTE2 [40] to hg19/1000 Genomes, and gene-wise p-values were calculated using Knowledge-Based mining system for Genome-wide Genetic studies (KGG2.5) [41]. This dataset was chosen because of its deep phenotyping and its theoretical consistency with findings from mouse experiments. The expression of the top-scoring genes in IASPSAD (*COL6A3*, *RYR3*, and *KLF12*) in mouse brain correlates with handling-induced convulsions, anxiety-like behavior, and acute functional tolerance to ethanol, respectively[33].

The Avon Longitudinal Study of Parents and Children (ALSPAC) GWAS gene-wise p-values were used to examine the ability of EW-dmGWAS to validate the EW-dmGWAS networks. This GWAS tested SNP association with a factor score calculated from 10 Alcohol Use Disorder Identification Test items for 4,304 (42.9% male) participants from Avon, UK. Samples were genotyped by the Illumina HumanHap550 quad genome-wide SNP platform [42]. This dataset was chosen because of its overall similarity to IASPSAD. Although the analyzed phenotypes were not identical between these two datasets, they were similar in that they both studied dependence symptoms, as opposed to non-diagnostic drinking measures. Additionally like IASPSAD, ALSPAC possessed the following important qualities: 100% of the sample was from the United Kingdom; the male to female ratio was roughly 1:1; SNPs were imputed to hg19/1000 Genomes; and gene-wise p-values were calculated by KGG2.5. No other GWAS dataset is as similar to IASPSAD to our knowledge, with respect to ancestral origin, genotyping, and phenotyping.

#### Protein network data

The Protein-Protein Interaction (PPI) network was obtained from the Protein Interaction Network Analysis (PINA 2.0) Platform (http://omics.bjcancer.org/pina/interactome.pina4ms.do). This platform was chosen because it includes PPI data from a wide array of databases, including: Intact, MINT, BioGRID, DIP, HPRD, and MIPS/Mpact. The *Homo sapiens* dataset was used for this analysis, due to it having much more content (166,776 binary interactions) than the mouse repository (only 13,865 binary interactions) [43, 44]. Uniprot IDs were used to match protein symbols to their corresponding gene symbols [45].

### Statistical methods

#### EW-dmGWAS

The edge-weighted dense module searching for GWAS (dmGWAS_3.0) R package was used to identify treatment-dependent edge-weighted modules (small, constituent networks) nested within the background network(s) of non-weighted, binary interactions (https://bioinfo.uth.edu/dmGWAS/). We used the PPI framework for the background network, IASPSAD GWAS gene-wise p-values [33] for the node-weights, and RMA values from acute ethanol- and saline-exposed mouse PFC, VTA, and NAc genomic data for edge-weights [25]. For the remainder of this manuscript, we will use the term “network” to refer to the background PPI framework, and “module” to refer to the resulting groups of interrelated genes nested within this larger network. By the EW-dmGWAS algorithm, higher node-weights represent lower (i.e. more significant) GWAS p-values, whereas higher edge-weights represent a greater difference in the correlation of two genes between ethanol and control groups. This is calculated by taking the difference of correlations in RMA expression values of the two genes in control vs. ethanol treated BXD lines. The module score algorithm incorporated edge- and node-weights, which were each weighted to prevent bias towards representation of nodes or edges in module score calculations. Such bias could cause some modules to be identified based almost solely on edge-weights or node-weights, as opposed to the two combined, which would defeat the purpose of integration. The respective weighting depends upon a parameter (λ) which is calculated prior to module searching, based on the entire set of node- and edge-weights and used across all module score calculations, as part of the EW-dmGWAS algorithm. Higher module scores thus represent higher edge- and node-weights. Genes were kept in a module if they increased the standardized module score (S_n_) by 0.5%. S_n_ corresponding to a permutation-based, empirical *q*_*FDR*_<0.05 were considered significant. A significant S_n_ (i.e. more significant *q*_*FDR*_ values) indicates that a module’s constituent genes are more highly associated with AD in humans, and their interactions with each other are more strongly perturbed by acute ethanol exposure in mice than randomly constructed modules of the same size.

Due to the redundancy of genes between modules, we modified the EW-dmGWAS output by iteratively merging significant modules that overlapped >80% until no modules had >80% overlap, for each brain region. Percent overlap represented the number of genes contained in both modules (for every possible pair) divided by the number of genes in the smaller module. We call the final resulting modules “mega-modules”. Standardized mega-module scores (MM-S_n_) were calculated using the algorithms employed by EW-dmGWAS. MM-S_n_ corresponding to *q*_*FDR*_<0.05 were considered significant (S1 Fig). Finally, connectivity (k) and Eigen-centrality (EC) were calculated using the igraph R package for each gene in each module to identify hub genes. Nodes with EC>0.2 and in the top quartile for connectivity for a module were considered to be hub genes.

#### Overlap with ALSPAC

Genes with an ALSPAC GWAS gene-wise *p*<0.001 were considered nominally significant, and will be referred to as “ALSPAC-nominal genes”. We used linear regression to test MM-S_n_’s prediction of mean ALSPAC GWAS gene-wise p-value of each mega-module. Given our hypothesis that EW-dmGWAS would identify alcohol-associated gene networks and prioritize them by association, we predicted that higher MM-S_n_’s would predict lower (i.e. more significant) mean GWAS p-values. Empirical p-values<0.017, reflecting Bonferroni correction for 3 independent tests (one per brain region): α = 0.05/3, were considered to represent significant association.

Overrepresentation of ALSPAC-nominal genes within each mega-module was analyzed for those modules containing >1 such gene. For each of these mega-modules, 10,000 modules containing the same number of genes were permuted to determine significance. Empirical p-values < 0.05/n (where n = total number of mega-modules tested) were considered significant.

#### Functional enrichment analysis

To determine if mega-modules with significant overrepresentation of ALSPAC-nominal genes represented an aggregation of functionally related genes, ToppGene (https://toppgene.cchmc.org/) was used to analyze functional enrichment. Categories of biological function, molecular function, cellular component, mouse phenotype, human phenotype, pathways, and drug interaction were tested for over-representation. All genes in the human genome were included in the reference gene set. This set was not limited to the ethanol-responsive genes included in this analysis, in order to preclude functional bias. Significant over-representation results were defined as p<0.01 (uncorrected), n≥3 genes overlap and n≤1000 genes per functional group. Given the number of categories and gene sets tested, our discussion below was narrowed to the most relevant categories, defined as Bonferroni-corrected *p*<0.1.

## Results

Of the initial 45,037 probesets for the mouse gene expression arrays, 16,131 were associated with human-mouse homologues and had *q*_*FDR*_<0.1 for ethanol responsiveness (S-score) in at least one of the three brain regions (Fig 1). These probesets corresponded to a total of 7,730 genes and were trimmed to a single probeset per gene by filtering for the most abundant probeset as described in Methods. After removing genes that were absent from either the PPI network or the IASPSAD dataset, the final background PPI network for EW-dmGWAS analysis contained 6,050 genes (nodes) and 30,497 interactions (edges). The nodes contained 25 of the 78 IASPSAD-nominal genes and 24 of the 100 ALSPAC-nominal genes. There was no overlap between the IASPSAD and ALSPAC nominal gene sets.

### Prefrontal cortex

**Fig 2:**
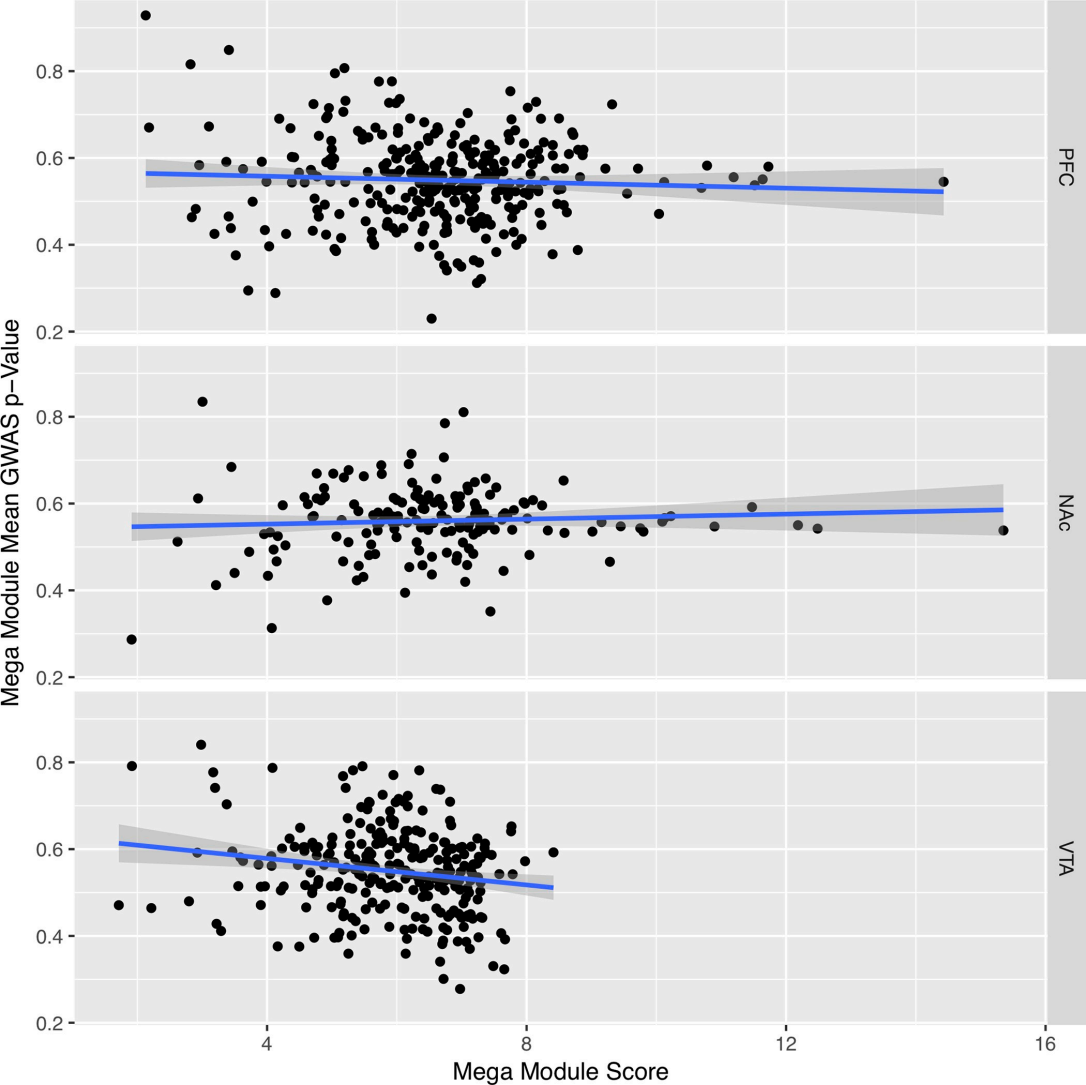
Mega module score v. module average ALSPAC GWAS p-Value. Correlation between each Mega Module’s score and average ALSPAC gene-wise GWAS p-value, for the Prefrontal Cortex (PFC) (β = -0.003, p = 0.327), Nucleus Accumbens (Nac) (β = 0.003, p = 0.390), and Ventral Tegmental Area (VTA) (β = -0.02, p = 0.003). Blue lines represent the line of best fit, estimated by linear regression, surrounded by their 95% confidence intervals (shaded gray).

For analysis using PFC expression data for edge-weights, results revealed 3,545 significant modules (*q*_*FDR*_<0.05) containing a total of 4,300 genes, with 14 ALSPAC-nominal genes and 18 IASPSAD-nominal genes. These modules were merged to form 314 mega-modules, all with significant MM-S_n_. Twelve mega-modules contained at least one ALSPAC-nominal gene, and 160 contained at least one IASPSAD-nominal gene. However, MM-S_n_ did not significantly predict mean ALSPAC GWAS gene-wise p-value (*β* = -0.003, *p* = 0.327, Fig 2).

**Fig 3:**
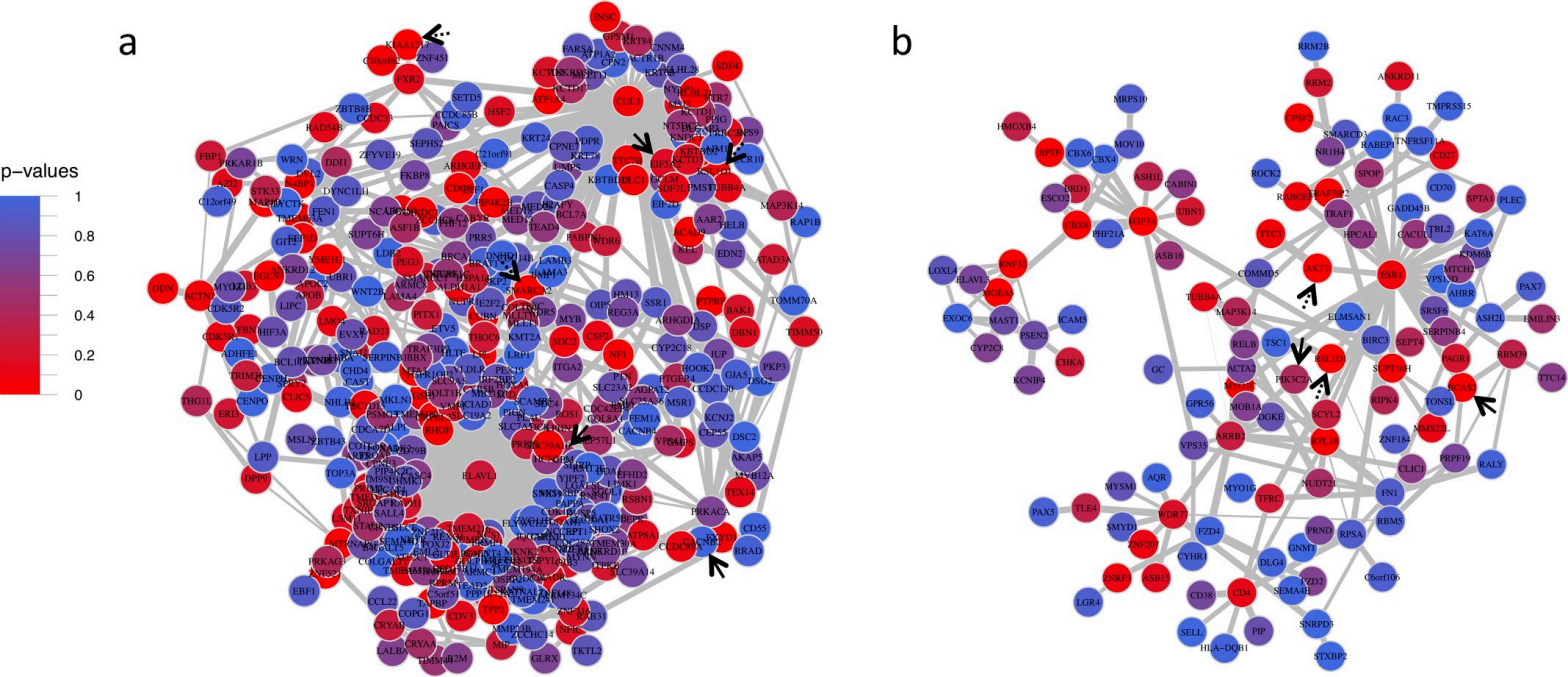
Prefrontal cortex mega modules aliceblue and cadetblue. Prefrontal Cortex Mega Modules Cadetblue (a) and Aliceblue (b). Solid black arrows point to ALSPAC GWAS nominal genes, and dotted black arrows represent IASPSAD nominal genes. Edge-width is proportional to the difference in correlation strength between treatment and control mice, and node color represents IASPSAD GWAS p-values.

Two mega-modules, Aliceblue and Cadetblue, contained multiple ALSPAC-nominal genes (Table 1). Because overrepresentation was tested for 2 mega-modules, *p*<0.025 (α = 0.05/2) was considered significant. Cadetblue, was significantly overrepresented with ALSPAC-nominal genes (Table 1). Each of Cadetblue’s ALSPAC- and IASPSAD-nominal genes was connected to one of its most highly connected hub genes, *ESR1* (estrogen receptor 1; connectivity (k) = 31, Eigen-centrality (EC) = 1) and *ARRB2* (beta-arrestin-2; k = 13, EC = 0.25) (Fig 3). Although the ALSPAC-nominal gene overrepresentation was not significant for Aliceblue, it approached significance (Table 1). Further, Aliceblue had the second-highest MM-S_n_ in the PFC and contained 3 ALSPAC-nominal genes and 3 IASPSAD-nominal genes (Table 1). For these reasons, Aliceblue was carried through to functional enrichment analysis. Aliceblue’s two hub genes were *ELAVL1* ((embryonic lethal, abnormal vision)-like 1; k = 165, EC = 1) and *CUL3* (cullin 3; k = 75, EC = 0.21), which were connected to two of the three ALSPAC-nominal genes. Of these, *CPM*’s (carboxypeptidase M’s) only edge was with *ELAVL1*, and *EIF5A2*’s (eukaryotic translation initiation factor 5A2’s) only edge was with *CUL3* (Fig 3).

**Table 1:** ALSPAC nominal gene overrepresentation.

Brain Region	Mega-modules	*k*_*g*_	MM-S_n_	MM-S_n_ *q*_*FDR*_	Overrep. *p*	Gene	IASPSAD GWAS *p*	ALSPAC GWAS *p*
PFC	aliceblue	392	11.19	<1E-16*	0.063	CPM	0.493	6.48E-05*
						CACNB2	0.978	4.97E-04*
						EIF5A2	0.163	8.06E-04*
						RSL1D1	3.48E-04*	0.217
						SMARCA2	4.91E-04*	0.877
						KIAA1217	8.84E-04*	0.904
	cadetblue	125	6.30	1.08E-06*	0.013*	BCAS2	0.029	4.65E-04*
						PIK3C2A	0.432	9.52E-04*
						RSL1D1	3.48E-04*	0.217
						AKT2	3.90E-05*	0.980
NAc	cadetblue2	195	8.04	8.06E-16*	0.042	CPM	0.493	6.48E-05*
						MGST3	0.358	4.62E-04*
	gray26	12	6.39	9.95E-11*	<0.001*	PCDH7	0.007	2.10E-04*
						BCAS2	0.029	4.65E-04*
VTA	coral	399	4.78	1.00E-06*	0.068	CPM	0.493	6.48E-05*
						DENND2C	0.018	4.33E-04*
						BIRC7	0.930	4.37E-04*
						MGST3	0.358	4.62E-04*
						PIK3CA	7.06E-05*	0.007
						TNN	3.00E-04*	0.018
						ANO6	6.32E-04*	0.780
						SMARCA2	4.91E-04*	0.877
						SIMC1	2.04E-04*	0.977
	limegreen	220	5.22	1.19E-07*	0.054	DENND2C	0.018	4.33E-04*
						EIF5A2	0.163	8.06E-04*
						RSL1D1	3.48E-04*	0.217
						CCND2	1.94E-04*	0.603
						AKT2	3.90E-05*	0.980
	bisque	89	6.22	7.57E-10*	0.006*	ACLY	0.701	2.21E-04*
						PRKG1	0.647	8.26E-04*
						AKT2	3.90E-05*	0.980

The following characteristics are displayed for each mega-module that contained >1 ALSPAC-nominal gene: affiliated brain region; total number of constituent genes (k_g_); constituent ALSPAC- and IASPSAD-nominal genes; empirical p-values for ALSPAC-nominal overrepresentation (Overrep. p); MM-S_n_,and the associated False Discovery Rate (MM-S_n_ qFDR).

* Significant *p*-values: *p*<0.05 for MM S_n_; *p*<0.05/n for ALSPAC overrepresentation, where n = number of tests per brain region; *p*<0.001 for IASPSAD and ALPSAC GWAS.

Both Cadetblue and Aliceblue showed significant enrichment in several functional categories (S3 Table). In sum, top functional enrichment categories for Aliceblue were related to actin-based movement, cardiac muscle signaling and action, increased triglyceride levels in mice, cell-cell and cell-extracellular matrix adhesion, and syndecan-2-mediated signaling. In contrast, Cadetblue’s top enrichment categories involved transcription-regulatory processes, specifically: RNA splicing, chromatin remodeling, protein alkylation and methylation, DNA replication regulation, several immune-related pathways, *NF-κβ* and Wnt signaling pathways, and reductase activity (Tables 2 and 3; S3 Table).

**Table 2:** Top gene ontology enrichment results for pfc mega module cadetblue.

Category	Name	p-value	q-value Bonferroni	Hit Count in Query List	Hit Count in Genome	Hit in Query List
GO: Biological Process	chromatin organization	1.50E-09	4.12E-06	23	776	SMYD1, ESR1, KAT6A, ASH1L, PAGR1, CBX4, KDM6B, ASH2L, MYSM1, PHF21A, BPTF, UBN1, CBX6, SUPT16H, SMARCD3, H3F3B, PAX5, PAX7, BRD1, CABIN1, MGEA5, NR1H4, CBX8
	histone modification	1.97E-06	5.40E-03	14	453	SMYD1, KAT6A, ASH1L, PAGR1, KDM6B, ASH2L, MYSM1, PHF21A, PAX5, PAX7, BRD1, MGEA5, NR1H4, CBX8
	covalent chromatin modification	2.87E-06	7.89E-03	14	468	SMYD1, KAT6A, ASH1L, PAGR1, KDM6B, ASH2L, MYSM1, PHF21A, PAX5, PAX7, BRD1, MGEA5, NR1H4, CBX8
	chromatin remodeling	1.47E-05	4.04E-02	8	165	SMYD1, ESR1, ASH2L, MYSM1, BPTF, SMARCD3, H3F3B, PAX7
	RNA splicing	1.60E-05	4.40E-02	12	403	SRSF6, NUDT21, BCAS2, RBM39, RALY, RBM5, PRPF19, AKT2, CPSF2, SNRPD3, WDR77, AQR
	protein alkylation	2.44E-05	6.71E-02	8	177	SMYD1, ASH1L, ASH2L, PAX5, PAX7, SNRPD3, WDR77, NR1H4
	protein methylation	2.44E-05	6.71E-02	8	177	SMYD1, ASH1L, ASH2L, PAX5, PAX7, SNRPD3, WDR77, NR1H4
GO: Cellular Component	nucleoplasm part	2.23E-05	7.49E-03	16	738	MMS22L, SRSF6, NUDT21, KAT6A, PAGR1, CBX4, ELMSAN1, ASH2L, RBM39, PHF21A, UBN1, TONSL, PRPF19, SPOP, CPSF2, BRD1
	chromosome	1.21E-04	4.07E-02	17	943	MMS22L, PSEN2, BCAS2, ESR1, KAT6A, ASH1L, ZNF207, ASH2L, ESCO2, CBX6, TONSL, SUPT16H, PRPF19, SMARCD3, H3F3B, NR1H4, CBX8
	ribonucleoside-diphosphate reductase complex	1.24E-04	4.17E-02	2	3	RRM2B, RRM2
	DNA replication factor A complex	1.39E-04	4.67E-02	3	16	BCAS2, TONSL, PRPF19
	nuclear replication fork	1.40E-04	4.71E-02	4	41	MMS22L, BCAS2, TONSL, PRPF19
	catalytic step 2 spliceosome	2.96E-04	9.94E-02	5	90	BCAS2, RALY, PRPF19, SNRPD3, AQR
GO: Molecular Function	oxidoreductase activity, acting on CH or CH2 groups	3.32E-05	1.62E-02	3	10	CYP2C8, RRM2B, RRM2
	oxidoreductase activity, acting on CH or CH2 groups, disulfide as acceptor	1.31E-04	6.38E-02	2	3	RRM2B, RRM2
	ribonucleoside-diphosphate reductase activity, thioredoxin disulfide as acceptor	1.31E-04	6.38E-02	2	3	RRM2B, RRM2
	ribonucleoside-diphosphate reductase activity	1.31E-04	6.38E-02	2	3	RRM2B, RRM2
	chromatin binding	1.69E-04	8.24E-02	12	516	ESR1, KAT6A, ASH1L, RELB, CBX4, KDM6B, ASH2L, PHF21A, TLE4, SMARCD3, H3F3B, CABIN1
Mouse Phenotype	increased immunoglobulin level	1.16E-06	2.92E-03	14	307	TRAF3IP2, GADD45B, SEMA4B, PSEN2, ESR1, SPTA1, ASH1L, BIRC3, RELB, MYSM1, CD4, PIK3C2A, RABGEF1, CABIN1
	abnormal humoral immune response	5.52E-06	1.39E-02	18	566	TRAF3IP2, GADD45B, SEMA4B, PSEN2, ESR1, SPTA1, MAP3K14, ASH1L, BIRC3, RELB, TNFRSF11A, MYSM1, CD4, PIK3C2A, CD38, RABGEF1, PAX5, CABIN1
	abnormal immunoglobulin level	7.68E-06	1.93E-02	17	522	TRAF3IP2, GADD45B, SEMA4B, PSEN2, ESR1, SPTA1, MAP3K14, ASH1L, BIRC3, RELB, TNFRSF11A, MYSM1, CD4, PIK3C2A, RABGEF1, PAX5, CABIN1
	increased IgG level	9.35E-06	2.35E-02	11	225	TRAF3IP2, GADD45B, SEMA4B, ESR1, SPTA1, ASH1L, BIRC3, MYSM1, CD4, PIK3C2A, CABIN1
	cortical renal glomerulopathies	1.18E-05	2.96E-02	10	188	TRAF3IP2, GADD45B, PSEN2, MYO1E, ESR1, SPTA1, RRM2B, ASH1L, RELB, PIK3C2A
	abnormal lymph node morphology	1.85E-05	4.66E-02	14	390	SELL, TRAF3IP2, TRAF1, PSEN2, ESR1, SPTA1, RRM2B, MAP3K14, BIRC3, RELB, TNFRSF11A, CD4, PIK3C2A, PIP
	glomerulonephritis	1.95E-05	4.91E-02	8	121	TRAF3IP2, GADD45B, PSEN2, ESR1, SPTA1, ASH1L, RELB, PIK3C2A
	abnormal B cell physiology	3.21E-05	8.07E-02	18	644	MYO1G, TRAF3IP2, GADD45B, SEMA4B, PSEN2, ESR1, SPTA1, MAP3K14, ASH1L, BIRC3, RELB, TNFRSF11A, MYSM1, CD4, PIK3C2A, RABGEF1, PAX5, CABIN1
Pathway	Signaling by Wnt	2.78E-06	2.47E-03	13	340	LGR4, ASH2L, FZD4, ARRB2, ZNRF3, TLE4, VPS35, H3F3B, AKT2, GNAO1, FZD2, MOV10, RAC3
	NF-kappa B signaling pathway	1.07E-04	9.44E-02	6	95	GADD45B, TRAF1, MAP3K14, BIRC3, RELB, TNFRSF11A
	Apoptosis	1.13E-04	9.97E-02	7	138	GADD45B, TRAF1, SEPT4, SPTA1, MAP3K14, BIRC3, AKT2

Functional enrichment results from ToppFun for Prefrontal Cortex Mega Module Cadetblue, where Bonferroni-corrected p<0.1.

**Table 3:** Top gene ontology enrichment results for pfc mega module aliceblue.

Category	Name	p-value	q-value Bonferroni	Hit Count in Query List	Hit Count in Genome	Hit in Query List
GO: Biological Process	regulation of actin filament-based movement	4.76E-08	2.07E-04	9	37	FXYD1, ATP1A2, DBN1, GJA5, JUP, KCNJ2, DSC2, DSG2, DSP
	cardiac muscle cell-cardiac muscle cell adhesion	7.53E-08	3.27E-04	5	7	CXADR, JUP, DSC2, DSG2, DSP
	regulation of cardiac muscle cell contraction	1.64E-07	7.11E-04	8	31	FXYD1, ATP1A2, GJA5, JUP, KCNJ2, DSC2, DSG2, DSP
	actin filament-based process	3.57E-07	1.55E-03	36	688	CDC42EP4, ACTN1, MYOZ1, MKLN1, FXYD1, RHOF, SDC4, CUL3, PRR5, CRYAA, ARHGDIA, ATP2C1, CCDC88A, STAU2, DYNLL1, DIXDC1, ATP1A2, CXADR, DBN1, PTGER4, GJA5, JUP, CDK5R1, NF1, KCNJ2, CACNB2, DSC2, DSG2, DSP, ARHGEF5, CASP4, LCP1, CSRP3, LIMK1, LDB3, LRP1
	cell communication involved in cardiac conduction	4.34E-07	1.89E-03	9	47	PRKACA, ATP1A2, CXADR, GJA5, JUP, CACNB2, DSC2, DSG2, DSP
	desmosome organization	8.59E-07	3.73E-03	5	10	SNAI2, JUP, DSG2, DSP, PKP3
	cardiac muscle cell action potential	1.07E-06	4.65E-03	9	52	ATP1A2, CXADR, GJA5, JUP, KCNJ2, CACNB2, DSC2, DSG2, DSP
	cardiac muscle cell contraction	1.07E-06	4.65E-03	9	52	FXYD1, ATP1A2, GJA5, JUP, KCNJ2, CACNB2, DSC2, DSG2, DSP
	bundle of His cell to Purkinje myocyte communication	1.55E-06	6.72E-03	5	11	GJA5, JUP, DSC2, DSG2, DSP
	regulation of cardiac muscle cell action potential	2.30E-06	9.99E-03	6	20	CXADR, GJA5, JUP, DSC2, DSG2, DSP
	bundle of His cell-Purkinje myocyte adhesion involved in cell communication	2.63E-06	1.14E-02	4	6	JUP, DSC2, DSG2, DSP
	regulation of heart rate by cardiac conduction	2.65E-06	1.15E-02	7	31	GJA5, JUP, KCNJ2, CACNB2, DSC2, DSG2, DSP
	cardiac conduction	3.37E-06	1.46E-02	13	131	FXYD1, PRKACA, ATP1A2, ATP1A4, CXADR, GJA5, JUP, KCNJ2, CACNB2, CACNB4, DSC2, DSG2, DSP
	cardiac muscle cell action potential involved in contraction	7.69E-06	3.34E-02	7	36	GJA5, JUP, KCNJ2, CACNB2, DSC2, DSG2, DSP
	regulation of actin filament-based process	1.05E-05	4.58E-02	21	343	CDC42EP4, FXYD1, SDC4, ARHGDIA, CCDC88A, STAU2, DIXDC1, ATP1A2, DBN1, PTGER4, GJA5, JUP, CDK5R1, KCNJ2, DSC2, DSG2, DSP, ARHGEF5, CSRP3, LIMK1, LRP1
	lipoprotein localization	1.34E-05	5.83E-02	5	16	APOB, APOC2, MSR1, CUBN, LRP1
	lipoprotein transport	1.34E-05	5.83E-02	5	16	APOB, APOC2, MSR1, CUBN, LRP1
	regulation of cardiac muscle contraction	1.36E-05	5.91E-02	9	70	FXYD1, PRKACA, ATP1A2, GJA5, JUP, KCNJ2, DSC2, DSG2, DSP
GO: Cellular Component	intercalated disc	2.90E-06	1.53E-03	9	59	ACTN1, ATP1A2, CXADR, GJA5, JUP, KCNJ2, DSC2, DSG2, DSP
	cell-cell contact zone	1.56E-05	8.21E-03	9	72	ACTN1, ATP1A2, CXADR, GJA5, JUP, KCNJ2, DSC2, DSG2, DSP
	desmosome	1.61E-04	8.49E-02	5	26	JUP, DSC2, DSG2, DSP, PKP3
GO: Molecular Function	protein binding involved in heterotypic cell-cell adhesion	8.62E-07	7.88E-04	5	10	CXADR, JUP, DSC2, DSG2, DSP
	protein binding involved in cell adhesion	1.15E-06	1.05E-03	6	18	CXADR, ITGA2, JUP, DSC2, DSG2, DSP
	protein binding involved in cell-cell adhesion	2.62E-06	2.39E-03	5	12	CXADR, JUP, DSC2, DSG2, DSP
	cell adhesive protein binding involved in bundle of His cell-Purkinje myocyte communication	2.64E-06	2.41E-03	4	6	JUP, DSC2, DSG2, DSP
Human Phenotype	Dilated cardiomyopathy	4.35E-05	3.89E-02	9	87	ACAD9, CRYAB, UBR1, JUP, DSG2, DSP, LAMA4, CSRP3, LDB3
	Right ventricular cardiomyopathy	8.82E-05	7.90E-02	4	13	JUP, DSC2, DSG2, DSP
Mouse Phenotype	increased circulating triglyceride level	1.27E-05	4.77E-02	16	179	ALPI, COL1A1, VLDLR, AGPAT2, WRN, APOB, APOC2, TXNIP, RSBN1, CSF2, PRKACA, BGLAP, MED13, LEPR, LIPC, LRP1
Pathway	Non-integrin membrane-ECM interactions	3.41E-05	4.72E-02	7	46	ACTN1, SDC2, SDC4, ITGA2, LAMA3, LAMA4, LAMB3
	Syndecan-2-mediated signaling events	4.44E-05	6.14E-02	6	33	SDC2, CSF2, PRKACA, ITGA2, NF1, LAMA3

Functional enrichment results from ToppFun for Prefrontal Cortex Mega Module Aliceblue, where Bonferroni-corrected p<0.1.

### Nucleus accumbens

Using NAc acute ethanol expression data for edge-weights yielded 3,460 significant modules containing a total of 4,213 genes, 15 of which were ALSPAC-nominal and 16 of which were IASPSAD-nominal. After merging by content similarity, there were 171 significant mega-modules. Nineteen MM contained at least one ALSPAC-nominal gene, and 73 MM contained at least one IASPSAD-nominal gene. However, MM S_n_ did not significantly predict MM mean ALSPAC GWAS gene-wise p-value (*β* = 0.003, *p* = 0.390). Two MMs, Cadetblue2 and Gray26, each contained two ALSPAC-nominal genes (Table 1). Because there were 2 tests for overrepresentation, *p*<0.025 (α = 0.05/2) was considered significant. Gray26, was significantly overrepresented with ALSPAC-nominal genes, and Cadetblue2 showed a trend towards overrepresentation with significance before correcting for multiple testing (Table 1).

**Fig 4:**
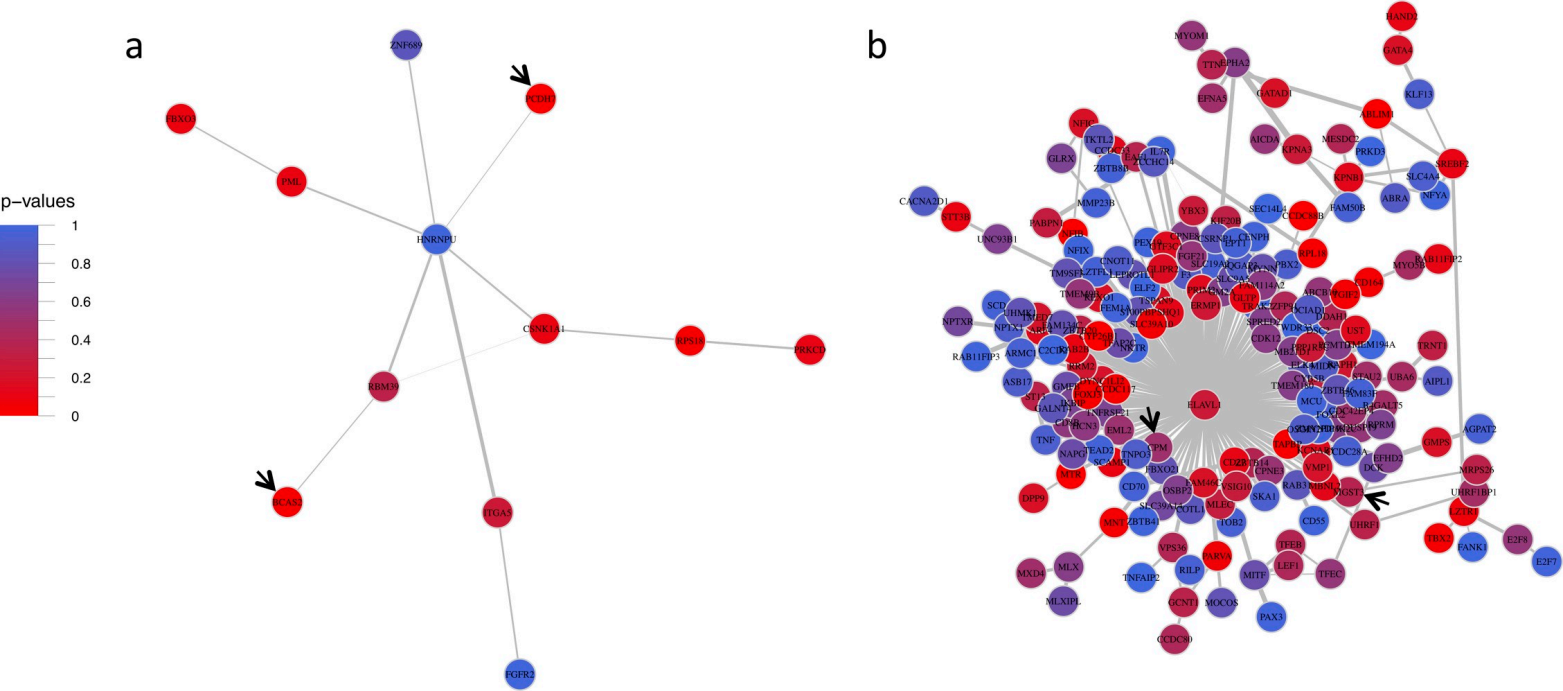
Nucleus accumbens mega modules Gray26 and Cadetblue2. Nucleus Accumbens Mega Modules Gray26 (a) and Cadetblue2 (b). Solid black arrows point to ALSPAC GWAS nominal genes. These modules did not contain IASPSAD nominal genes. Edge-width is proportional to the difference in correlation strength treatment and control mice, and node color represents IASPSAD GWAS p-values.

Gray26’s most central hub gene was *HNRNPU* (heterogeneous nuclear ribonucleoprotein U; connectivity = 6, Eigen-centrality = 1), followed by *RBM39* (RNA binding motif protein 39; k = 3, EC = 0.46) and *CSNK1A1* (k = 3, EC = 0.37). The two ALSPAC-nominal genes *BCAS2* (breast carcinoma amplified sequence 2) and *PCDH7* (protocadherin 7), shared their only edges with *RBM39* and *HNRPNPU*, respectively (Fig 4A). As seen in the PFC’s Aliceblue, *EAVL1* was a hub gene of Cadetblue2. *ELAVL1* (k = 136, EC = 1) was connected to both of the ALSPAC-nominal genes, and served as the only connection for *CPM* and one of two connections for *MGST3* (microsomal glutathione S-transferase 3) (Fig 4B). Strikingly, PFC Aliceblue and NAc Cadetblue 2 showed a highly significant overlap in their gene content, with 72 overlapping genes (S2 Table; p = 2.2 x 10^−16^).

Both Cadetblue2 and Gray26 were significantly enriched with several functional categories (S3 Table). Like PFC Cadetblue, NAc Cadetblue2 was functionally enriched for gene groups related to nuclear function with transcription regulation pathways, particularly those involving RNA polymerase activity. Gray26 was most significantly enriched with genes related to functions involving: telomere maintenance, organelle organization, ribonucleoprotein complexes, and syndecan-mediated signaling (Tables 4 and 5; S3 Table).

**Table 4:** Top gene ontology enrichment results for nucleus accumbens mega module cadetblue2.

Category	Name	p-value	q-value Bonferroni	Hit Count in Query List	Hit Count in Genome	Hit in Query List
GO: Biological Process	negative regulation of transcription from RNA polymerase II promoter	9.38E-06	2.93E-02	23	810	TGIF2, ZBTB20, SREBF2, E2F7, FOXL2, NFIB, NFIC, NFIX, MITF, MNT, TBX2, MLX, YBX3, TFAP2C, MXD4, E2F8, ZBTB14, MLXIPL, UHRF1, TNF, ELK4, PAX3, LEF1
GO: Molecular Function	RNA polymerase II transcription factor activity, sequence-specific DNA binding	1.80E-09	1.20E-06	27	678	ZBTB20, SREBF2, GATA4, E2F7, CSRNP1, FOXL2, NFIB, NFIC, NFIX, MITF, NFYA, MNT, HAND2, TBX2, TFEB, TEAD2, MLX, YBX3, FOXJ3, TFAP2C, E2F8, MLXIPL, KLF13, ELF2, ELK4, PAX3, LEF1
	transcriptional repressor activity, RNA polymerase II transcription regulatory region sequence-specific binding	3.04E-06	2.03E-03	11	182	ZBTB20, SREBF2, E2F7, MITF, MNT, TBX2, MLX, YBX3, TFAP2C, E2F8, MLXIPL
	transcription factor activity, RNA polymerase II core promoter proximal region sequence-specific binding	6.11E-06	4.08E-03	15	365	ZBTB20, SREBF2, FOXL2, NFIB, NFIC, MITF, NFYA, HAND2, TBX2, TFEB, TFAP2C, E2F8, MLXIPL, KLF13, LEF1
	RNA polymerase II regulatory region sequence-specific DNA binding	8.95E-06	5.98E-03	20	632	SREBF2, GATA4, E2F7, FOXL2, NFIB, NFIC, NFIX, MITF, NFYA, MNT, HAND2, TBX2, TFEB, MLX, YBX3, TFAP2C, E2F8, MLXIPL, KLF13, LEF1
	transcription regulatory region DNA binding	9.52E-06	6.36E-03	24	862	SREBF2, GATA4, E2F7, FOXL2, NFIB, NFIC, NFIX, MITF, NFYA, MNT, HAND2, TBX2, TFEB, MLX, YBX3, TFAP2C, E2F8, ZBTB14, MLXIPL, KLF13, UHRF1, TNF, ELK4, LEF1
	regulatory region DNA binding	1.01E-05	6.74E-03	24	865	SREBF2, GATA4, E2F7, FOXL2, NFIB, NFIC, NFIX, MITF, NFYA, MNT, HAND2, TBX2, TFEB, MLX, YBX3, TFAP2C, E2F8, ZBTB14, MLXIPL, KLF13, UHRF1, TNF, ELK4, LEF1
	RNA polymerase II regulatory region DNA binding	1.03E-05	6.87E-03	20	638	SREBF2, GATA4, E2F7, FOXL2, NFIB, NFIC, NFIX, MITF, NFYA, MNT, HAND2, TBX2, TFEB, MLX, YBX3, TFAP2C, E2F8, MLXIPL, KLF13, LEF1
	regulatory region nucleic acid binding	1.07E-05	7.14E-03	24	868	SREBF2, GATA4, E2F7, FOXL2, NFIB, NFIC, NFIX, MITF, NFYA, MNT, HAND2, TBX2, TFEB, MLX, YBX3, TFAP2C, E2F8, ZBTB14, MLXIPL, KLF13, UHRF1, TNF, ELK4, LEF1
	transcription regulatory region sequence-specific DNA binding	1.32E-05	8.82E-03	21	705	SREBF2, GATA4, E2F7, FOXL2, NFIB, NFIC, NFIX, MITF, NFYA, MNT, HAND2, TBX2, TFEB, MLX, YBX3, TFAP2C, E2F8, MLXIPL, KLF13, UHRF1, LEF1
	sequence-specific double-stranded DNA binding	2.50E-05	1.67E-02	21	736	SREBF2, GATA4, E2F7, FOXL2, NFIB, NFIC, NFIX, MITF, NFYA, MNT, HAND2, TBX2, TFEB, MLX, YBX3, TFAP2C, E2F8, MLXIPL, KLF13, UHRF1, LEF1
	core promoter proximal region sequence-specific DNA binding	7.08E-05	4.73E-02	14	399	SREBF2, GATA4, FOXL2, NFIB, NFIC, MITF, NFYA, TBX2, TFEB, E2F8, MLXIPL, KLF13, UHRF1, LEF1
	core promoter proximal region DNA binding	7.47E-05	4.99E-02	14	401	SREBF2, GATA4, FOXL2, NFIB, NFIC, MITF, NFYA, TBX2, TFEB, E2F8, MLXIPL, KLF13, UHRF1, LEF1
	transcriptional activator activity, RNA polymerase II transcription regulatory region sequence-specific binding	9.15E-05	6.11E-02	13	358	GATA4, CSRNP1, FOXL2, NFIB, NFIC, NFIX, MITF, NFYA, HAND2, TFEB, TFAP2C, KLF13, LEF1
	double-stranded DNA binding	1.25E-04	8.37E-02	21	824	SREBF2, GATA4, E2F7, FOXL2, NFIB, NFIC, NFIX, MITF, NFYA, MNT, HAND2, TBX2, TFEB, MLX, YBX3, TFAP2C, E2F8, MLXIPL, KLF13, UHRF1, LEF1
Human Phenotype	Synophrys	3.61E-05	2.06E-02	5	48	ZBTB20, NFIX, MITF, KLF13, PAX3
Mouse Phenotype	absent coat pigmentation	2.38E-05	6.28E-02	4	15	MITF, TFEB, TFEC, PAX3

Functional enrichment results from ToppFun for Nucleus Accumbens Mega Module Cadetblue2, where Bonferroni-corrected p<0.1.

**Table 5:** Top gene ontology enrichment results for nucleus accumbens mega module Gray26.

Category	Name	p-value	q-value Bonferroni	Hit Count in Query List	Hit Count in Genome	Hit in Query List
GO: Biological Process	negative regulation of telomere maintenance via telomerase	2.46E-05	2.92E-02	2	12	HNRNPU, PML
	negative regulation of organelle organization	4.65E-05	5.52E-02	4	340	PRKCD, FGFR2, HNRNPU, PML
	negative regulation of telomere maintenance via telomere lengthening	5.06E-05	6.00E-02	2	17	HNRNPU, PML
GO: Cellular Component	ribonucleoprotein complex	8.99E-04	8.99E-02	4	751	CSNK1A1, RPS18, BCAS2, HNRNPU
	intracellular ribonucleoprotein complex	8.99E-04	8.99E-02	4	751	CSNK1A1, RPS18, BCAS2, HNRNPU
Pathway	Syndecan-4-mediated signaling events	2.67E-04	7.44E-02	2	31	PRKCD, ITGA5
	Syndecan-2-mediated signaling events	3.03E-04	8.44E-02	2	33	PRKCD, ITGA5

Functional enrichment results from ToppFun for Nucleus Accumbens Mega Module Gray26, where Bonferroni-corrected p<0.1.

### Ventral tegmental area

Use of VTA control/ethanol gene expression responses for edge weighting initially resulted in 3,519 significant modules containing a total of 4,188 genes in EW-dmGWAS analysis. Merging by content similarity, resulted in 276 MMs, each with a significant MM S_n_. Seventeen ALSPAC-nominal genes and 19 IASPSAD-nominal genes were spread across 25 and 156 mega-modules, respectively. Furthermore, MM-S_n_ significantly predicted mean ALSPAC GWAS gene-wise *p*-value (*β* = -0.02, *p* = 0.003).

**Fig 5:**
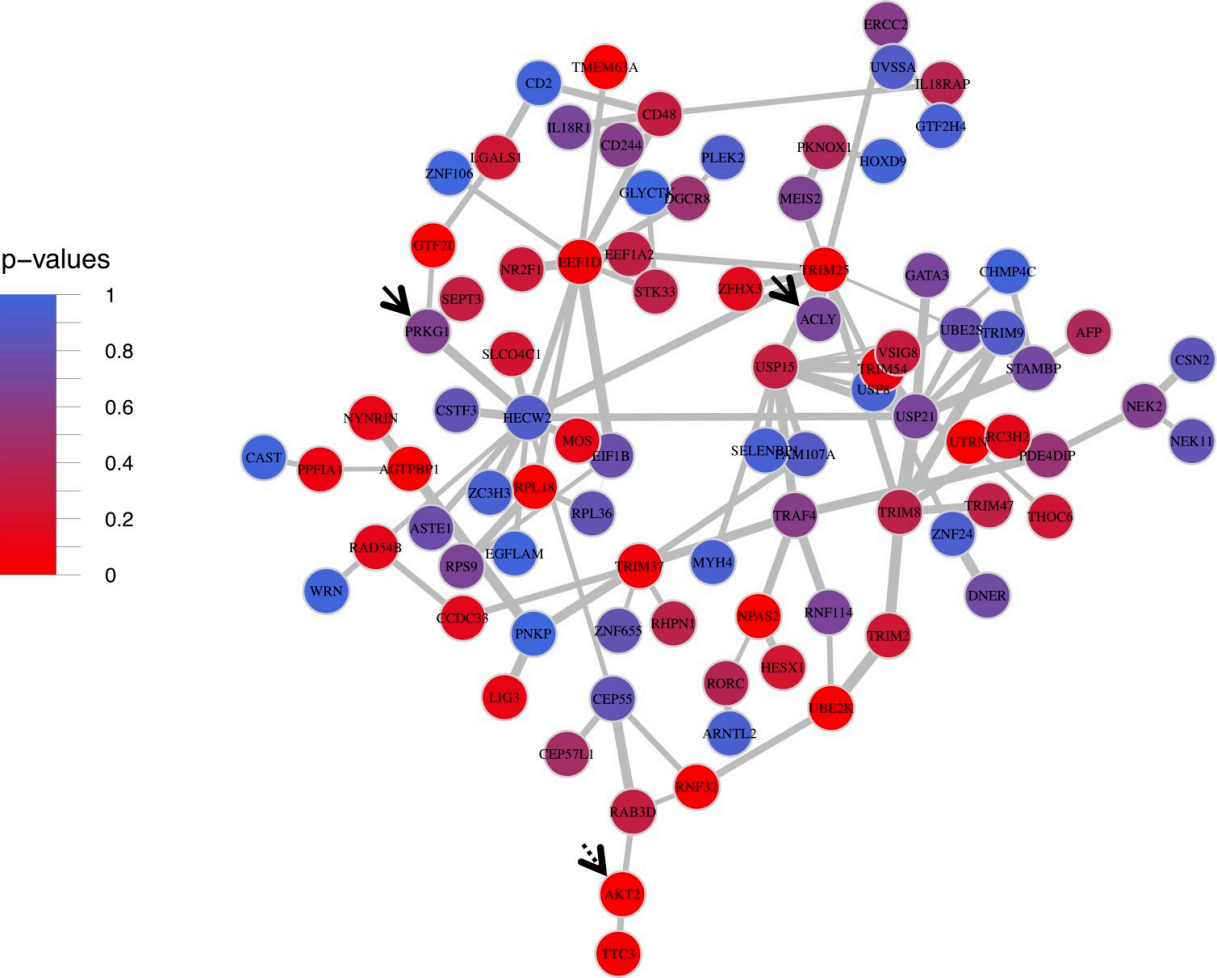
Ventral tegmental area mega module bisque. Ventral Tegmental Area Mega Modules Bisque. Solid black arrows point to ALSPAC GWAS nominal genes, and dotted black arrows represent IASPSAD nominal genes. Edge-width is proportional to the difference in correlation strength between treatment and control mice, and node color represents IASPSAD GWAS p-values.

Mega-modules with the highest representation of ALSPAC-nominal genes included Coral, Limegreen, and Bisque (Table 1). Because there were 3 tests for overrepresentation, *p*<0.017 (α = 0.05/3) was considered significant. Although overrepresentation of ALSPAC-nominal genes was not significant in Coral and Limegreen, it was significant in Bisque, which has the highest MM-S_n_ of the three (Table 1; Fig 5). Bisque contained four highly interconnected genes: *USP21* (ubiquitin specific peptidase 21; k = 10, EC = 1), *USP15* (ubiquitin specific peptidase 15; k = 10, EC = 0.65), *TRIM25* (tripartite motif-containing 25; k = 10, EC = 0.49), and *HECW2* (HECT, C2 and WW domain containing E3 ubiquitin protein ligase 2; k = 12, EC = 0.48). *HECW2* and *TRIM25* shared edges with this MM’s IASPSAD-nominal genes *PRKG1* (protein kinase, cGMP-dependent, type I) and *ACLY* (ATP citrate lyase), respectively. However, none of the hub genes shared an edge with Bisque’s ALSPAC nominal gene, *AKT2* (AKT serine/threonine kinase 2). Finally, Bisque had significant enrichment in several functional categories (S3 Table). It was most significantly enriched with genes associated with ubiquitination, ligase and helicase activity, and eukaryotic translation elongation (Table 6; S3 Table).

**Table 6:** Top gene ontology enrichment results for ventral tegmental area mega module bisque.

Category	Name	p-value	q-value Bonferroni	Hit Count in Query List	Hit Count in Genome	Hit in Query List
GO: Cellular Component	nucleolus	6.41E-07	1.24E-04	17	894	ZNF106, NEK2, EEF1D, RPL36, PNKP, SELENBP1, ZNF655, RPS9, WRN, GATA3, ZFHX3, RORC, DGCR8, TTC3, ARNTL2, NEK11, RPL18
	eukaryotic translation elongation factor 1 complex	1.27E-04	2.47E-02	2	4	EEF1D, EEF1A2
GO: Molecular Function	ubiquitin-protein transferase activity	4.98E-07	1.33E-04	12	414	RC3H2, TRAF4, UBE2K, TRIM2, TRIM25, TRIM9, HECW2, TRIM8, UBE2S, RNF114, TTC3, TRIM37
	ubiquitin-like protein transferase activity	9.70E-07	2.59E-04	12	441	RC3H2, TRAF4, UBE2K, TRIM2, TRIM25, TRIM9, HECW2, TRIM8, UBE2S, RNF114, TTC3, TRIM37
	acid-amino acid ligase activity	3.42E-06	9.12E-04	9	259	RC3H2, TRIM2, TRIM25, TRIM9, HECW2, TRIM8, RNF114, TTC3, TRIM37
	ligase activity, forming carbon-nitrogen bonds	9.78E-06	2.61E-03	9	295	RC3H2, TRIM2, TRIM25, TRIM9, HECW2, TRIM8, RNF114, TTC3, TRIM37
	tubulin-glycine ligase activity	1.87E-05	5.00E-03	8	244	RC3H2, TRIM2, TRIM9, HECW2, TRIM8, RNF114, TTC3, TRIM37
	protein-glycine ligase activity	1.87E-05	5.00E-03	8	244	RC3H2, TRIM2, TRIM9, HECW2, TRIM8, RNF114, TTC3, TRIM37
	protein-glycine ligase activity, initiating	1.87E-05	5.00E-03	8	244	RC3H2, TRIM2, TRIM9, HECW2, TRIM8, RNF114, TTC3, TRIM37
	coenzyme F420-0 gamma-glutamyl ligase activity	1.87E-05	5.00E-03	8	244	RC3H2, TRIM2, TRIM9, HECW2, TRIM8, RNF114, TTC3, TRIM37
	ribosomal S6-glutamic acid ligase activity	1.87E-05	5.00E-03	8	244	RC3H2, TRIM2, TRIM9, HECW2, TRIM8, RNF114, TTC3, TRIM37
	coenzyme F420-2 alpha-glutamyl ligase activity	1.87E-05	5.00E-03	8	244	RC3H2, TRIM2, TRIM9, HECW2, TRIM8, RNF114, TTC3, TRIM37
	UDP-N-acetylmuramoylalanyl-D-glutamyl-2,6-diaminopimelate-D-alanyl-D-alanine ligase activity	1.87E-05	5.00E-03	8	244	RC3H2, TRIM2, TRIM9, HECW2, TRIM8, RNF114, TTC3, TRIM37
	protein-glycine ligase activity, elongating	1.87E-05	5.00E-03	8	244	RC3H2, TRIM2, TRIM9, HECW2, TRIM8, RNF114, TTC3, TRIM37
	tubulin-glutamic acid ligase activity	2.05E-05	5.46E-03	8	247	RC3H2, TRIM2, TRIM9, HECW2, TRIM8, RNF114, TTC3, TRIM37
	protein-glutamic acid ligase activity	2.17E-05	5.79E-03	8	249	RC3H2, TRIM2, TRIM9, HECW2, TRIM8, RNF114, TTC3, TRIM37
	ligase activity	2.38E-05	6.35E-03	10	415	LIG3, RC3H2, TRIM2, TRIM25, TRIM9, HECW2, TRIM8, RNF114, TTC3, TRIM37
	DNA helicase activity	2.43E-04	6.49E-02	4	65	ERCC2, GTF2H4, RAD54B, WRN
Pathway	Eukaryotic Translation Elongation	1.67E-04	8.37E-02	5	98	EEF1D, RPL36, RPS9, EEF1A2, RPL18

Functional enrichment results from ToppFun for Ventral Tegmental Area Mega Module Bisque, where Bonferroni-corrected p<0.1.

## Discussion

To our knowledge, this is the first study to directly co-analyze human GWAS with mouse brain ethanol-responsive gene expression data to identify ethanol-related gene networks relevant to AD. Unlike previous studies that have employed cross-species validation methods for specific genes or gene sets, this study analyzed human and mouse data in tandem to identify gene networks across the entire genome, using the EW-dmGWAS algorithm. This approach successfully identified significantly ethanol-responsive and AD-associated gene networks, or modules. We further improved the existing EW-dmGWAS algorithm by merging highly redundant modules to create more parsimonious mega-modules, thus decreasing complexity without sacrificing significance. Additionally, we validated these results by testing for overrepresentation with, and mega-module score prediction by, signals from an independent GWAS dataset. Overall, our findings suggest that such direct integration of model organism expression data with human protein interaction and GWAS data can productively leverage these data sources. Furthermore, we present initial evidence for novel, cross-validated gene networks warranting further study for mechanisms underlying AUD.

### Identification of network-level associations across GWAS datasets

One major concern with existing GWAS studies on AD had been the relative lack of replication across studies. Although some very large GWAS studies on alcohol consumption have shown replicable results [13–15], those do not account for all previously identified associations. We reasoned that our integrative gene network-querying approach might identify networks that shared signals from different GWASs on AD, even if the signals were not from the same genes across GWASs. Concordant with this hypothesis, VTA mega-module scores significantly predicted average gene-wise p-values from an independent GWAS dataset, ALSPAC (Fig 2). This suggests that ethanol-responsive gene expression networks in this brain region may be particularly sensitive to genetic variance and thus are highly relevant to mechanisms contributing to genetic risk for AD. This is possibly attributable to the involvement of VTA dopaminergic reward pathways in the development of AD [46]. Further investigation of dopaminergic neuronal response to acute ethanol administration, and the association between this response and proclivity for developing dependence is needed.

Although scores did not prioritize mega-modules with respect to ALSPAC results in PFC and NAc, individual mega-modules were overrepresented with ALSPAC signals (Table 1). The ALSPAC-overrepresented VTA and PFC mega-modules also contained nominally significant genes from the GWAS dataset used for the network analysis, IASPSAD. These results suggest that the integration of acute ethanol-related expression data from mice and human PPI can identify functional networks that associate signals from different GWAS datasets.

### Composition and structure of mega-modules

Functional composition of mega-modules varied between brain regions for the most part. For example, although Aliceblue (PFC) and Cadetblue2 (NAc) shared the hub gene *ELAVL1*, ALSPAC-nominal gene *CPM*, and had a significant overlap in their gene content, their functional enrichment results were very different (Tables 3 and 4). These results suggest that brain regional ethanol-responsive gene expression results likely had an important impact on composition of networks, thus leveraging protein-protein interaction network information and GWAS results.

Despite such differences, the mega-modules presented in Table 1 shared certain structural similarities. Most of the IAPSAD- and ALSPAC-nominal genes in these modules shared edges with hub genes (Figs 3–5). These hub genes included: *CUL3 and ELAVL1* from PFC Aliceblue; *ESR1* from PFC Cadetblue; *ELAVL1*rom NAc Cadetblue2; *TRIM25* and *HECW2* from VTA Bisque. Further, GWAS nominally significant genes (IASPAD or ALSPAC) generally were not hub genes in the derived networks (see Figs 3–5; S2 Table). This may be consistent with the general tenet that genetic variation in complex traits does not produce major alterations in cellular function, but rather modulation of cellular mechanisms for maintaining homeostasis. Hub genes may be more functionally more closely related to a given trait, but likely have such widespread influence so as to be evolutionarily resistant to genetic variation in complex traits. This is also consistent with the hypothesis that omnigenic influences are an important feature of complex traits such as AUD [47].

One hub gene was found to influence network structure in both PFC and NAc. *ELAVL1* is a broadly expressed gene that acts as a RNA-binding protein in AU-rich domains, generally localized within 3’-UTRs of mRNA. As such, *ELAVL1* has been shown to alter mRNA stability by altering binding of miRNA or other factors influencing mRNA degradation [48] and has been implicated in activity-dependent regulation of gene expression in the brain with drug abuse [49]. The large interaction space for *ELAVL1* in PFC Alice Blue and NAc Cadetblue 2 and the multiple nominal GWAS hits within these genes suggest that *ELAVL1* could have an important modulatory function on the network of genes susceptible to genetic variation in AUD.

### Functional aspects of mega-modules

This theory regarding network structure is further supported by our functional enrichment analysis, which revealed several small groups of functionally related genes within each mega-module. All of the mega-modules discussed above (Table 1) contained at least one GWAS-nominal gene in the top enrichment groups, except Cadetblue2, which still had GWAS-nominal genes in its significant enrichment groups (S3 Table).

Another unifying feature across these mega-modules, except Aliceblue, was significant functional enrichment for pathways that regulate gene expression. Specifically, these pathways were related to chromatin organization, RNA splicing, and translation- and transcription-related processes (S3 Table). This is not surprising, as alterations in gene expression have long been proposed as a mechanism underlying long-term neuroplasticity resulting in ethanol-dependent behavioral changes, and eventually dependence [50].

In contrast, the largest functional enrichment groups unique to Aliceblue were related to actin-based filaments and cardiac function (Tables 2 and 3). Actin not only provides cytoskeletal structure to neurons, but also functions in dendritic remodeling in neuronal plasticity, which likely contributes to AD development [51, 52]. Aliceblue was also significantly enriched for the syndecan-2 signaling pathway, and contained the *SDC2* gene itself, which functions in dendritic structural changes together with F-actin [53]. Additionally, the most significant enrichment group unique to Cadetblue was the Wnt signaling pathway, which also regulates actin function [54, 55]. Of note, a prior study has shown that *ARRB2* (a Cadetblue hub gene and member of Wnt signaling pathway) knockout rats display significantly decreased levels of voluntary ethanol consumption and psychomotor stimulation in response to ethanol [56]. These findings highlight the potential importance of postsynaptic actin-related signaling and dendritic plasticity in PFC gene networks responding to acute ethanol and contributing to genetic risk for AD. Future studies may aim to confirm this association by investigating changes in actin and dendritic processes in response to acute ethanol exposure, and whether or not the degree of these changes is associated with development of dependence.

Finally, although the NAc Cadetblue2 mega-module was highly enriched for functions related to transcriptional regulation, it also contained the gene *FGF21* within its interaction space (S2 Table and Fig 4B). FGF21 is a member of the fibroblast growth factor gene family and is a macronutrient responsive gene largely expressed in liver. Importantly FGF21 has been shown to be released from the liver by ethanol consumption and negatively regulates ethanol consumption by interaction with brain FGF-receptor/beta-Klotho complexes. Beta-Klotho, a product of the *KLB* gene, is an obligate partner of the FGF receptor and has recently been shown to have a highly significant association with alcohol consumption in recent very large GWAS studies [14, 15]. Although the role of *FGF21* and *KLB* in AD are not currently known, the association of *FGF21* with the Cadetblue2 mega-module, containing nominally associated genes from AD GWAS studies, is a possible additional validation of the utility of our studies integrating protein-protein interaction information (tissue non-specific), AD GWAS (tissue non-specific) and brain ethanol-responsive gene expression.

### Potential weaknesses and future studies

The studies presented here provide evidence for the utility of integrating genomic expression data with protein-protein interaction networks and GWAS data in order to gain a better understanding of the genetic architecture of complex traits, such as AD. Our analysis also generated several testable hypotheses regarding gene networks and signaling mechanisms related to ethanol action and genetic burden for AD. However, these studies utilized acute ethanol-related expression data in attempting to identify mechanisms of AD, a chronic ethanol exposure disease. Use of a chronic exposure model could provide for a more robust integration of the expression data and GWAS signals. However, we feel the current study is valid, since acute responses to ethanol have been repeatedly shown to be a heritable risk factor for AD [57–59]. Further, large GWAS studies have recently shown significant genetic correlation and overlapping significant genes between alcohol consumption and alcohol dependence phenotypes [60, 61]. We have also recently demonstrated a very high degree of overlap in mouse brain expression changes between acute ethanol exposure and a chronic ethanol vapor exposure model thought to mimic aspects of alcohol dependence [30]. Our laboratory has also recently reported that an acute ethanol-responsive gene network from the same microarray data used for studies in this manuscript showed significant association, at a network level, with AD in data from the COGA GWAS analysis of AD [31]. Finally, the cross-species analysis of acute ethanol responses and AD allowed us to explore networks involved in specific brain regional initial response to ethanol that are also related to dependence. Therefore, our findings may have implications for mechanistic activations or changes occurring upon initial ethanol exposure, and ultimately contributing to the development of dependence.

A potential shortcoming for this work regards the limited size of the GWAS studies utilized and differences in phenotypic assessment. The IASPSAD study was based on AD diagnosis, whereas ALSPAC was based on a symptom factor score. Had we used larger GWAS studies based on the same assessment criteria, it is possible that greater overlap of GWAS signals within mega-modules would have been observed. Recent large GWAS studies on ethanol have, to date, generally concerned measures of ethanol consumption, rather than a diagnosis of alcohol dependence per se [14, 15]. For this reason, we focused this initial effort on GWAS studies concerned with alcohol dependence. However, using the IASPAD and ALSPAC studies allowed us to identify gene networks that are robust across both the severe end of the phenotypic spectrum (i.e. diagnosable AD), and for symptoms at the sub-diagnostic level.

Overall, this analysis successfully identified novel ethanol-responsive, AD-associated, functionally enriched gene expression networks in the brain that may play a causal role in the developmental pathway from first ethanol exposure to AD. This is the first analysis to identify such networks by directly co-analyzing brain regional gene expression data, protein-protein interaction data, and GWAS summary statistics. The identified modules provided insight into common pathways between differing signals from independent, largely underpowered, yet deeply phenotyped GWAS datasets. This supports the conjecture that the integration of different GWAS results at a gene network level, rather than simply looking for replication of individual gene signals, could make use of previously underpowered datasets and identify common genetic mechanisms relevant to AD. Future expansion of such approaches to integrate additional model organism chronic ethanol-responsive gene sets with the rapidly evolving GWAS literature on alcohol consumption and dependence, together with validation of key targets by gene targeting in animals models, may provide both novel insight for the neurobiology of AD and the development of improved therapeutic approaches.

## Supporting information

S1 Fig Analytical pipeline of steps following EW-dmGWAS. Empirical p-values were calculated from standardized module scores based on a Z-distribution. The original EW-dmGWAS module score, permutation, and score standardization algorithms were used to calculate the respective Mega Modules parameters. Modules were considered to have >80% overlap if >80% of the genes in the smaller module was contained in the larger module. False Discovery Rates were calculated based on the Benjamini-Hochberg algorithm, using the “stats” package in R. Intramodular connectivity was defined as the number of edges (i.e. connections) attached to that node (i.e. gene). Eigen-Centrality was calculated using the “igraph” package in R.
(PDF)

S1 Table Brain region-specific S-score values. One table per brain region, containing each of the following values: RMA values and S-scores from the maximally expressed probeset per gene, for each BXD strain; the associated probeset IDs, human gene symbols, and mouse gene symbols; and the Fisher’s combined False Discovery Rate (q-value) for each probeset.
(XLSX)

S2 Table Mega module characteristics. One table per brain region, containing each of the following characteristics, for all significant Mega Modules: name; constituent genes; ALPSAC and IASPSAD p-values for each gene; Mega Module score (S_n_), p-value (S_n__p), and False Discovery Rate (S_n__qFDR); and intramodular eigencentrality and connectivity. Significance values < 10^−16^ are rounded to 0.
(XLSX)

S3 Table Mega module gene ontology enrichment. One table for each ALSPAC-overrepresented Mega Module, containing ToppFun output for gene ontology enrichment groups with *p*<0.01 and minimum group size of 3 genes and maximum size of 1,000 genes, for the following categories: Biological Process, Cellular Component, Molecular Function, Human Phenotype, Mouse Phenotype, and Pathways.
(XLSX)
