# Supplementary figures and images for: Cross-species alcohol dependence-associated gene networks: Co-analysis of mouse brain gene expression and human genome-wide association data
Source: PLoS One. 2019 Apr 24;14(4):e0202063. doi: 10.1371/journal.pone.0202063 (PMC6481773; doi:10.1371/journal.pone.0202063)

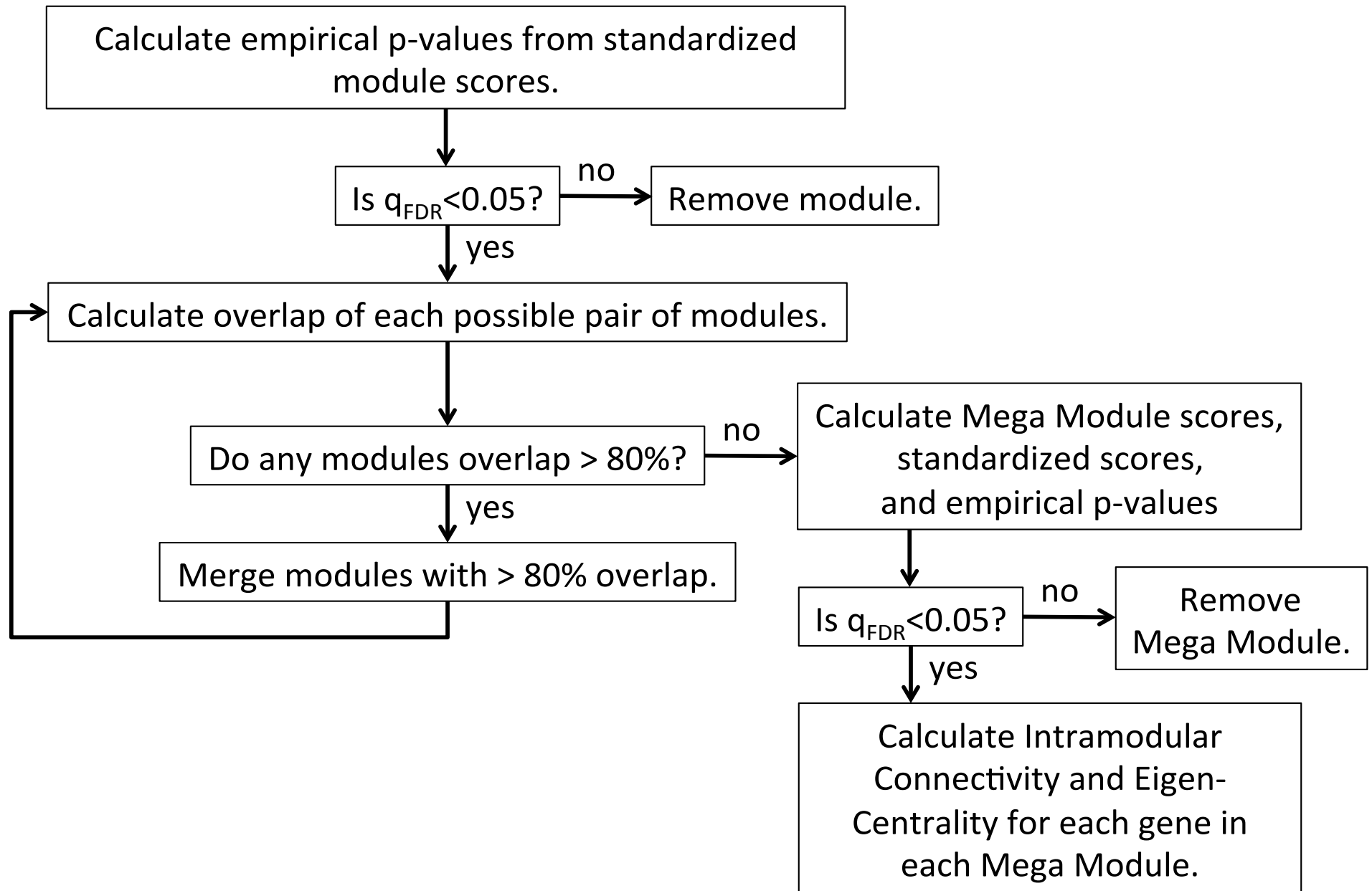

Supplement: S1 Fig — Empirical p-values were calculated from standardized module scores based on a Z-distribution. The original EW-dmGWAS module score, permutation, and score standardization algorithms were used to calculate the respective Mega Modules parameters. Modules were considered to have >80% overlap if >80% of the genes in the smaller module was contained in the larger module. False Discovery Rates were calculated based on the Benjamini-Hochberg algorithm, using the “stats” package in R. Intramodular connectivity was defined as the number of edges (i.e. connections) attached to that node (i.e. gene). Eigen-Centrality was calculated using the “igraph” package in R. (PDF) [file pone.0202063.s001.pdf]
